# Observational Data for Integrated Maternity Care: Experiences with a Data-Infrastructure for Parents and Children in the Netherlands

**DOI:** 10.5334/ijic.7012

**Published:** 2023-12-20

**Authors:** Zoë T. M. Scheefhals, Eline F. de Vries, Joyce M. Molenaar, Mattijs E. Numans, Jeroen N. Struijs

**Affiliations:** 1 Department of National Health and Healthcare, National Institute for Public Health and the Environment (RIVM), The Netherlands; 2 Department of Public Health and Primary Care, LUMC Health Campus The Hague, The Netherlands; 3 Department of Health Economics and Healthcare, National Institute for Public Health and the Environment (RIVM), The Netherlands

**Keywords:** observational data, routinely collected data, health policy, integrated care, integrated maternity care, data-infrastructure

## Abstract

**Introduction:**

Observational data are increasingly seen as a valuable source for integrated care research. Especially since the growing availability of routinely collected data and quasi-experimental methods. The aim of this paper is to describe the potentials and challenges when using observational data for integrated maternity care research, based on our experience from developing and working with the Data-InfrAstructure for ParEnts and childRen (DIAPER).

**Methods and Results:**

We provide a description of DIAPER, which is a linked data-infrastructure on the individual level based on maternity care claims data, quality and utilization of maternity care and data from municipal registries, covering the life course from preconception to adulthood. We then discuss potentials and practical applications of DIAPER such as to evaluate alternative payment models for integrated maternity care, to set the policy agenda regarding postpartum care, to provide insights into value of care and into provider variation, and to evaluate (policy) interventions designed to promote and support integrated maternity care. This is relevant for several stakeholders: policy makers, payers, providers and clients/patients. Based on experiences with DIAPER, we identify remaining challenges: missing data sources (especially self-reported outcomes), suboptimal quality of data, privacy concerns and potential biases introduced during data linkage, and describe how these challenges were tackled within the applications of DIAPER.

**Conclusions:**

With DIAPER we demonstrated that using observational data can be of added value for integrated care research, but also that challenges remain. It is essential to keep exploring and developing the possibilities of observational data and continue the discussions in the scientific community. Learning from each other’s successes and failures will be critical.

## Introduction

The knowledge base on health and diseases derived from randomized controlled trials (RCTs) has strongly contributed to the design and implementation of complex models for integrated care delivery and consequently to policies that have shaped our healthcare and public health systems [1]. Research based on data gathered in RCTs is still considered as the ‘golden standard’ due to their unique ability to strictly control treatment conditions for a well-defined study population and maximize compliance and internal validity [1,2,3,4]. At the same time, observational studies come with severe methodological challenges, especially when they are based on routinely collected healthcare data: the conditions in this type of study are far less controlled and nonrandomized, weakening internal validity and making it easy for confounding to occur. However, RCTs may not always be applicable – or translatable – to real world practice, or results may not always be generalizable outside of the sample. This is also the case in integrated care settings, which are generally too complex and diverse for RCT-type studies to be feasible. For this reason, observational data can be of substantial value to integrated care research. Not only for the evaluation of policy changes and interventions, and their effect on health outcomes, but also to supply the system with data and predictions on expected costs and resource use [5].

A major benefit of observational data is that they reflect real world situations, potentially leading to ‘real life’ results with strong external validity [3,6,7]. The nature of most observational data is that they are routinely collected from the general population and they often cover a large majority of the population. Therefore, observational data can be used to evaluate health policies that are implemented in complex systems, for which RCTs are not feasible or ethical. Furthermore, the use of existing and readily available observational data is inherently cost- and time-saving as the data have already been collected during daily work and re-use might reduce the administrative burden of providers by saving double work. This is in line with the principles for FAIR data (Findable, Accessible, Interoperable, Reusable) [8]. By supporting the interoperability and reusability of data, the burden for professionals and patients can potentially be decreased while also creating richer data-infrastructures.

Because of its inherent ability to provide real world results, observational studies can complement RCTs. For example, by studying the effects of interventions in larger and more diverse populations or for longer follow-up periods to confirm or extend the effects observed in an RCT (e.g., to study the long-term effects of a vaccine or drug in the general population) [3,4]. Moreover, observational studies provide an opportunity to yield results where RCTs are not feasible or ethical [9], as is the case when evaluating health policies focused on integrated maternity care, or many other interventions that are implemented in complex settings.

In addition, the use of observational data is particularly relevant when studying integrated maternity care as the vast majority of pregnant women consults more than one maternity care provider during pregnancy and delivery [10,11]. When evaluating the quality of care provided by complex care models including multiple maternity care providers, monodisciplinary datasets do not suffice. Linked observational datasets in combination with the origination of quasi-experimental designs [12,13] can be of substantial added value to strengthen the research on integrated (maternity) care, by allowing researchers to study the multidisciplinary, integrated care network as a whole and by enabling them to compare the quality of care between networks and providers [3,4,14,15,16].

At the same time, several challenges of observational data have been overcome. Up until recently, many observational datasets were small and lacked diversity by mostly including small samples from subpopulations [17,18]. Moreover, most observational datasets did not capture all key components of care related to the target populations. The need for ‘richer’ population-based registers which capture all relevant key components including health outcomes, healthcare use, claims data, data on quality and the wider determinants of health (among which housing and living conditions, unemployment and welfare, access to health and social care services and education), is increasingly acknowledged and turned into action over the past years [17,19,20,21]. It has been proposed that these richer data-infrastructures can lead to a better understanding of populations’ health, their risk factors and patterns of care [14,22]. In the Netherlands, we have aimed to develop such a richer nationwide data-infrastructure based on observational data sources to study integrated maternity care.

In this article, we discuss the potentials and remaining challenges of observational data for integrated care research based on our experience from developing and working with DIAPER (Data-InfrAstructure for ParEnts and childRen). Specifically, we provide a description of DIAPER and review the potentials and challenges based on practical applications of DIAPER for several stakeholders (i.e., policy makers, payers, providers and clients/patients). We address various points of consideration that could be relevant to other researchers facing similar challenges as we expect that the availability of routinely collected observational data will increase at an enormous pace across the globe.

## The data-infrastructure for parents and children (DIAPER) explained

In Dutch maternity care, information on many variables at the individual pregnancy level is routinely registered nationwide by healthcare professionals, such as midwives, nurses and obstetricians. These data sources can provide relevant insights and can be used to inform policy makers, payers and professionals on how to (further) improve the quality of care and health outcomes for patients through integrated care and similar initiatives. This is the aim of our Dutch nationwide parent-child linked data-infrastructure that we named ‘DIAPER’. DIAPER combines data from several existing data sources including data from municipalities (e.g., nationality, ethnicity, marital status, household composition), data from the National Tax Authority (e.g. (household) income, capital and property), data on education, data from perinatal registries on maternity care use and health outcomes and nationwide claims data. These data sources cover a vast majority (>98%) of all pregnancies in the Netherlands and currently include data on over 3.5 million pregnancies (including characteristics of the mothers, fathers and children) from 2000 to 2020 (on-going virtual birth cohort).

### DIAPER and the wider determinants of health

**Figure 1:**
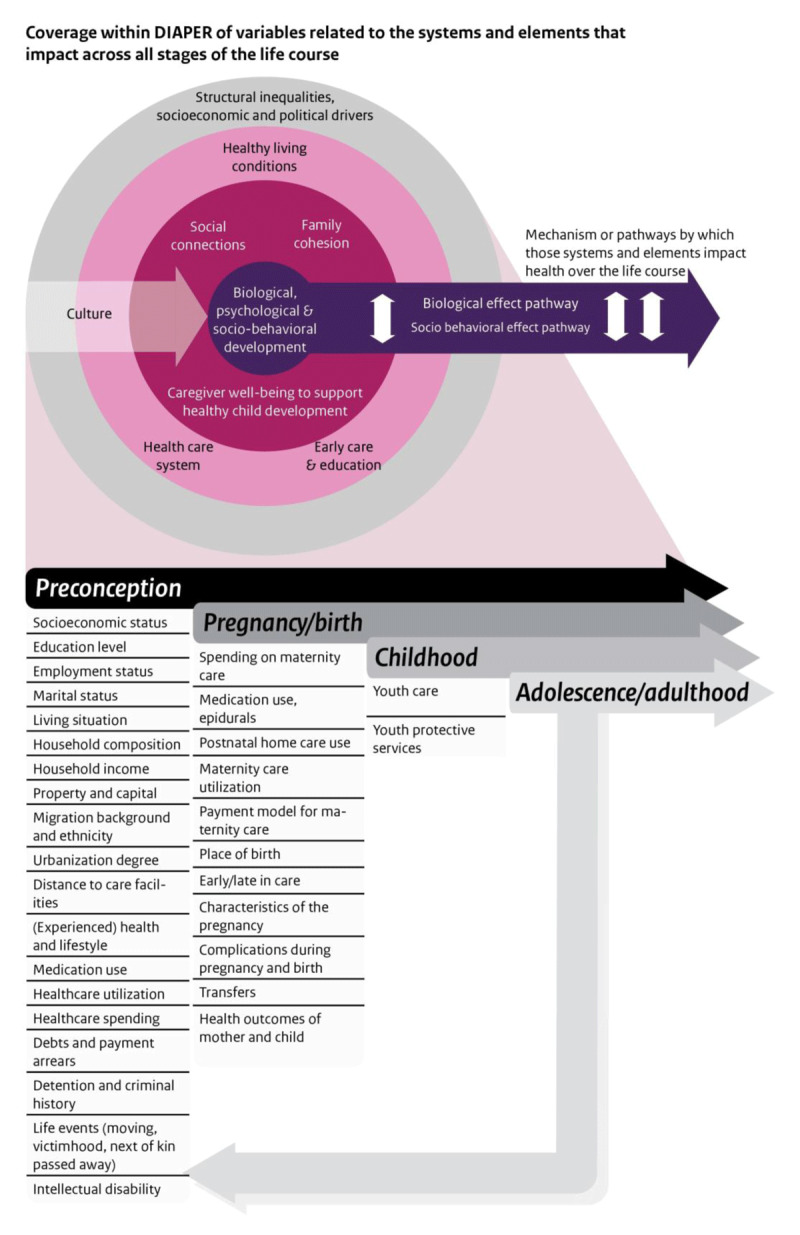
Schematic presentation of the variables in DIAPER within the Vibrant and Healthy Kids conceptual framework [23].

There are many factors at play during the preconception period, pregnancy, birth and early life that set the odds for individuals during their childhood, adolescence and adulthood [23]. These factors have intergenerational effects for individuals and subsequently affect the next generation of children as well. Examples of these systems and elements are the socioeconomic and political setting, living conditions, early care and education, the healthcare system, social connections and family cohesion. The data within DIAPER cover the full range of these different systems and elements, as outlined in Figure 1. Consequently, DIAPER facilitates analyses for a wide variety of research questions by incorporating data from both the medical and social domain. DIAPER was developed to study integrated maternity care and early life, because these topics are high on the policy agenda due to relatively high perinatal mortality rates in the Netherlands in the past [24,25] and the complex issue of social inequalities originating in early childhood [26].

### Data sources

**Figure 2:**
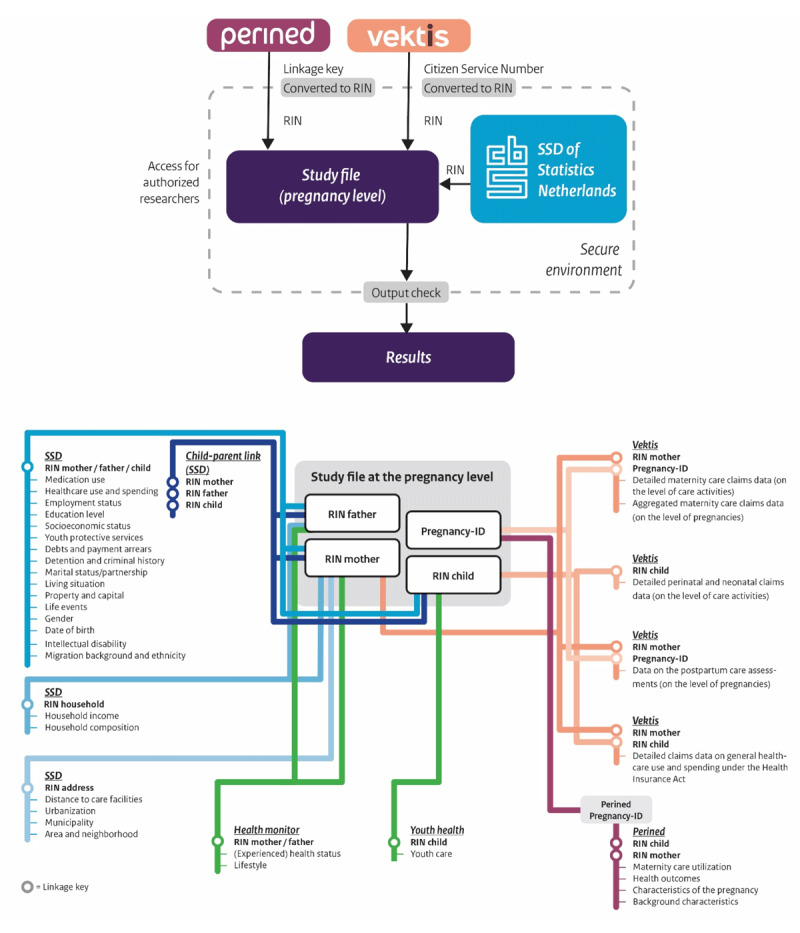
Overview of DIAPER’s data sources and the available files and variables including employed linkage key.

DIAPER is a dynamic infrastructure in which data from different sources can be combined to create a variety of different study populations for different research purposes. The sample size, structure and contents depend on the project, research question and the included data sources. Currently, the three main sources of data in DIAPER are: Perined, Vektis and Statistics Netherlands (depicted in Figure 2).

Perined is the Dutch Perinatal Registry and provides nationwide data on maternity care use and health outcomes of mothers and children [27]. Its aim is to improve the quality of perinatal care through the monitoring and registration of care processes and outcomes. The national registry contains routinely collected data on 96% of all deliveries in the Netherlands [28]. Data on pregnancy, delivery and neonatology are available on an individual level (mother, child and pregnancy) from 1999 till now. This includes data on type of care provided, maternal and perinatal health outcomes and background characteristics. The Perined data offer possibilities for monitoring and comparison and are useful for researchers, healthcare professionals as well as policy makers.

Vektis is the Dutch healthcare information center and provides routinely collected claims data from all Dutch health insurers on maternity and perinatal care use and spending [29,30]. The aim of Vektis is to collect claims data from all Dutch health insurers and to use these data to provide insight in healthcare utilization, spending and quality. Vektis is primarily used for the system of risk equalization in the Netherlands [31]. Because of the nature of the Dutch Health Insurance Act (mandatory basic insurance for all Dutch citizens) and the commitment of all Dutch health insurers to upload their data (to participate in the risk equalization system), Vektis covers roughly 99% of the Dutch (insured) population [29]. Aside from using the data to provide insights themselves, Vektis also started offering customized information services for external researchers in 2004. This usually concerns individual data on an aggregated level (e.g., total spending on GP care per year), but data on a more detailed level can be requested for specific research projects and topics. Such detailed claims data on maternity care are available, and included in DIAPER, as of 2015.

Statistics Netherlands (SN) provides access to their System of Social statistical Datasets (SSD) [32,33]. This wealth of linkable data (microdata) provides information on nearly 20 themes (including health and welfare, income and spending, labor and social security, population, and education) and contributes to the extensive coverage of sociodemographic characteristics within DIAPER. The data in the SSD are originally provided by a variety of (government) organizations (e.g., the National Tax Authority, municipalities, the Civil Service for Road Traffic (RDW)). SN transforms crude data into harmonized and linkable datasets. They also take care of pseudonymizing the data. In this process, unique persons are identified based on (a combination of) identifying variables and a unique ‘Record Identification Number’ (RIN) is assigned to each person in the data. This procedure is often easy and not prone to error (e.g., when Citizen Service Numbers are available), but can sometimes be more difficult. For example, in the case of the Perined data where a combination of several variables (date of birth mother/child, postal code 6 digits, sex of child and multiple pregnancy yes/no) has to be used as an identifier in order to assign a RIN. The RIN is the identifier through which different datasets can be linked. In addition, SN provides a highly safeguarded Remote Access (RA) platform in order to safely manage, analyze and store data, syntaxes and output [34]. DIAPER is located on this platform. Before any results can be exported out of this secure environment, an output check is performed by SN to prevent unwanted disclosures and protect the privacy of everyone involved. As DIAPER only consists of linked, secondary data, no additional ethical approval is required for DIAPER itself. Researchers working with DIAPER have to adhere to the requirements of SN and the other relevant data providers.

More details on the alignment of DIAPER with the principles for FAIR data can be found in Supplement 1.

### Linkage process

The data files within DIAPER provide data on different levels (e.g., pregnancy level, child level, parent(s) level, household level, address level). In order to properly link all relevant data, these different data files need to be transformed, harmonized and linked to each other in the appropriate order and on the correct linkage keys and levels. A simplified illustration of this procedure can be found in Figure 2, as well as more details on the different data files.

In a nutshell, this process consists of the following steps. Firstly, all relevant data from the SSD are harmonized and linked at the individual level of mothers, fathers and children. Secondly, the different Vektis data files are converted to the same level (i.e., the pregnancy level) and linked. After this, all that takes place around one pregnancy can be brought together in the data by grouping all claims related to one pregnancy and relabeling them. Thirdly, the Perined data can be linked to the Vektis data based on the pregnancy-ID (i.e., a unique identifier for each pregnancy of a mother). Finally, the Perined-Vektis combined data can be enriched by linking the relevant SSD data about mothers, fathers and children to the study file on the pregnancy level. Supplement 2 shows the results of the linkage procedure. Background characteristics of the population in DIAPER were compared to those registered by SN (the official national registry) and were comparable, giving no suggestion that the population in DIAPER is a selective sample of the entire population (see S2). This is an important indicator for the quality of the data and for the implications of the results derived from DIAPER.

## Current applications of DIAPER

**Figure 3:**
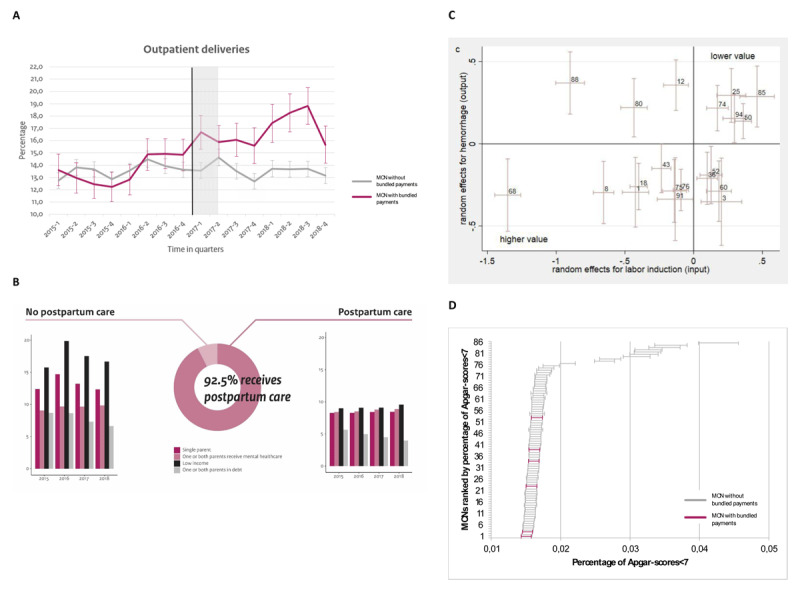
Current applications of DIAPER **(A-B-C-D)**.

To demonstrate the various potentials of observational data for integrated care and its value to policy makers, payers, providers and clients/patients, we will discuss some of DIAPER’s current applications as illustrated in Figure 3 (ABCD). We categorized these applications based on the stakeholder for whom it is most relevant in our opinion. Intuitively, most of these examples are relevant for more than one type of stakeholder.

### For policy makers

DIAPER serves as the quantitative base for various evaluations of Dutch policies with respect to integrated maternity care. Policy makers can use the information derived from these evaluations to decide whether and how to proceed. For example, DIAPER was used to evaluate an experiment with a bundled payment model for Dutch maternity care that was launched in six regions in 2017. The effects of the bundled payment model on healthcare utilization, health outcomes and healthcare spending were measured using a difference-in-differences (DiD) design [35] (Scheefhals et al., submitted). This quasi-experimental method mimics an experimental research design (where a real one is not feasible) using observational data, allowing researchers to properly evaluate complex policy interventions such as those focused on integrated care. We were able to define a highly comparable control group with respect to age, ethnicity, urbanization degree (obtained from SN), gestational age and mode of delivery (obtained from Perined). Figure 3A demonstrates crude data for the outcome (rate of outpatient deliveries) of the experiment group (pink) and the control group (grey). The results of these analyses have been presented to policy makers, but also to payers and maternity care providers to provide them with insights in how care utilization patterns, the level of integration and collaboration between providers, and the quality of maternity care are affected by the introduction of an alternative payment model that is designed to enhance integrated maternity care. This project is a good illustration of how observational data can be employed to provide real world evidence on policies that are implemented in a complex integrated care setting. The results and implications of these analyses serve as a tool to discuss and determine the future of the bundled payment model in Dutch maternity care.

Furthermore, DIAPER is used as a means to gain insight into postpartum care (in Dutch: kraamzorg) utilization in the Netherlands and subsequently, for agenda setting regarding postpartum care policies and integrated collaboration between postpartum care providers and other providers in the medical and social domain. Postpartum care provision at home plays an important role in providing each newborn with a healthy and promising start in life and can also benefit the rest of the household [36,37]. Research shows that people with a lower socioeconomic status use postpartum care less often, which is associated with higher healthcare expenditures for mother and child after delivery [36]. A break-down of postpartum care utilization, according to background characteristics (such as income, household composition, debts, mental healthcare use) as an indication of potential (health) inequalities between different groups in society, can be found in Figure 3B. This shows that 7.5% of eligible households did not receive postpartum care in 2015–2018. Within this group the percentage of households with a lower income or a single parent is higher than in the group that did receive postpartum care. The results of this serve as relevant input for the Dutch policy agenda to adjust policies, both in the medical and social domain, and pay more attention to certain groups that do not receive the care they need. Integrated collaboration and coordination regarding this issue can boost improvements.

### For payers

DIAPER has relevant contributions for payers, like insurance companies representing clients, or patients, because it can measure both spending and quality. Therefore, DIAPER can provide insights into issues regarding value in care. In the Netherlands, this is particularly relevant as Dutch health insurers are tasked with supervising and promoting the quality of care [31,38,39]. An example of this is the assessment we did to explore whether Dutch Maternity Care Networks (MCNs) (regional networks of providers to shape integrated maternity care) show associations of low-value services and maternal and neonatal health outcomes (De Vries et al., under review). This is, again, a good example of how observational data sources can be used to provide real world evidence while also being cost and time-efficient. We used neonatal mortality, NICU-admission, Apgar-score <7, hemorrhage post-partum and perineum damage as outcomes and caesarean section, spontaneous delivery and epidural analgesia as low-value care indicators. We found substantial variation across MCNs and were able to get a grasp on what defines value (in part) as we found persistent results across MCNs for the low-value care indicators. Figure 3C shows the MCN performance on the value-indicator ‘hemorrhage after labor induction’. MCNs at the bottom left show higher value as they perform relatively less low-value services and have less adverse outcomes (relative to other MCNs). Yet, despite access to many case-mix variables, we still detected variance within MCNs, which may have clouded our results. Insight into the value of care within and across MCNs is important for payers (i.e., health insurers) to monitor the progress towards value, for providers to make budget allocation decisions and for both payers and providers for the development of alternative payment models.

### For providers

An example of how DIAPER can be applied to benefit providers (like hospitals, midwives, general practitioners, nurses or obstetricians) is a project in which we benchmarked MCNs (regional networks of providers to shape integrated maternity care) and looked at the regional variation in the registered Apgar-score (a measure for the health of the child immediately after birth [40]). For this analysis, we have adjusted the Apgar-scores (obtained from Perined) for case-mix variables from the Perined and SN data, such as the age of the mother and the urbanization degree. The different regions have been arranged based on their percentage of Apgar-scores below 7 (this is an indication that the child needs medical attention) in Figure 3D. A positive aspect of this project is that the data that we used for the analysis is routinely collected by the care providers themselves in the primary care process. The purpose of this analysis was to explore whether there is room for quality improvements in MCNs and whether there are opportunities for regions to learn from each other. These results can provide useful insights for providers and integrated care networks as a starting point for quality improvements (e.g., as an outset to draw up quality improvement agreements or for prioritizing quality improvement initiatives) and they can also be relevant input for the discussions between providers and payers.

### For clients/patients

As may have become clear from the descriptions above, the results of the analyses based on DIAPER are not directly aimed at informing the general population of clients/patients. The information is not immediately applicable for patients to make informed decisions in daily care practice. However, the knowledge derived from DIAPER does contribute to improving the quality of care and the care experience of future mothers and families by providing policy makers, payers and providers with the necessary information and tools to improve quality and value in care and with insights on the status of integrated maternity care.

## Remaining challenges and opportunities

Regardless of the recent progress made in the field of reused routinely collected healthcare data and other observational data, several challenges still remain with respect to missing data, quality of the data, privacy and turning data into meaningful reports. These challenges provide opportunities for further improvement of observational studies and minimizing their limitations for integrated care research.

Despite the fact that there is already a wide variety of data sources available within DIAPER, some important data sources are still missing. DIAPER, for example, lacks data from the majority of municipal child and youth care organizations (in Dutch: JGZ, *jeudgezondheidszorg*) and from the NCO [41] on education in the Netherlands. In addition, efforts are made to create broad, standardized Patient Reported Outcome Measures (PROMs) and Patient Reported Experience Measures (PREMs) to collect data that can be included in nationwide observational data sources and that can improve the ability to fully capture the concept of value in care, including the softer measures, and to get a better grasp of the progress and results of integrated care in the context of maternity care.

Another challenge is that the quality of any observational data-infrastructure largely depends on the quality of the individual data sources and the diligence with which they are shared, harmonized and linked. The data within DIAPER is predominantly registered in the primary care process and its application for research purposes is its secondary use. While this is cost and time-saving, it also makes for mistakes to be a common occurrence. It is crucial that the data registration is carried out completely and correctly as mistakes in this step are irreversible. Several learning and improvement cycles are essential to improve and assure the desired level of quality of the registered data, as Perined has carried out for example. Also Vektis is continuously working to improve the quality of the registration and the transfer of the data. SN has its own built in system to verify and improve the quality of the registered data. For the data files provided by SN, we can rely on their quality checks. For the additional data, provided by Perined and Vektis and uploaded on our request to the SN platform, we are more involved in the quality assurance process and are in constant contact with Perined and Vektis on this topic. As researchers using secondary data, it is important to make a proper assessment of the data quality but also consider the role we want to play in improving its quality. In some cases, the same data is available in different sources and we can chose the most reliable source. For example, in the two example applications described above in which we benchmarked MCNs and looked at low-value care indicators across MCNs, we had the option to use the MCN classification (i.e., to which MCN is a pregnancy assigned in the data) from either Vektis or Perined. We opted for the Perined classification as they are in close contact with the MCNs and their data is therefore expected to be more complete and more reliable (with regards to the classification).

Moreover, in regard to the linkage process of the different data files, the risk of errors is high and this can potentially introduce bias, which can disproportionately affect disadvantaged groups [42]. In the case of DIAPER, this issue arises for example with the incomplete registration of stillborn babies both in the perinatal and the municipal registries, which causes a potential selection effect during the linkage process. Other examples of types of bias that are common in maternity and perinatal epidemiology, and that researchers should be apprehensive of, are fixed cohort bias, live-birth bias, immortal time bias and season of conception as a confounder [43].

Furthermore privacy concerns need to be considered. With increasing possibilities, there are growing societal concerns with regards to the use of personal data. Especially since the technical possibilities are expected to increase even further in the coming years, it is important to determine now where the boundaries should be set. In response to these concerns, advanced methods of data protection are increasingly developed but also new technologies are developed which address these privacy concerns. Federated learning is an example of such a new technology which allows for decentralized analysis of harmonized local data sources without physically transferring any data. Calculations are performed locally without sharing the privacy-sensitive data itself. Initiatives employing federated learning to pregnancy and childhood cohorts are already on the rise [44].

Finally, we face the complex challenge of turning data into valuable information applicable to daily practice. The value of linked, routinely collected observational data has not definitively been proven yet, for the aims mentioned in this paper, and we should strive to foster and demonstrate its added value in the long run. Current described applications of DIAPER are diverse and worthwhile, but can still be enhanced to generate even more ‘useful reports’ that address the needs and wishes of policy makers, payers, providers and clients/patients more effectively.

## Conclusion

We aimed to demonstrate through DIAPER the added value of using observational data for the field of integrated care and beyond. Nationwide individual level data-infrastructures such as DIAPER could be developed in many other disciplines within and outside the healthcare sector where large, routinely collected data sources are already available but hardly utilized for research purposes. During the process of developing and working with DIAPER we have encountered several challenges, such as selective missing data and privacy concerns, and have explored ways to overcome them that can be relevant for other researchers taking on similar endeavors.

Based on our experience with DIAPER, we discussed several applications for policy makers, payers, providers and clients/patients, and we identified potentials (providing real world evidence, cost- and time-saving for professionals) and challenges (missing data, suboptimal quality of data, privacy concerns, potential biases).

To conclude, the additional value of using routinely collected observational data for integrated care and health policy evaluations is considerable. Existing data-infrastructures such as DIAPER exemplify potentials, but also point out the remaining challenges. It is essential to keep exploring and developing the possibilities of observational data. As the use of observational data continues to grow, learning from each other’s successes and failures will be critical.

## Additional File

The additional file for this article can be found as follows:

10.5334/ijic.7012.s1 Supporting information. Appendix with detailed information on 1) alignment with FAIR principles; 2) checks for selectivity within DIAPER and 3) the codebook of DIAPER.
