# Supplementary material for: Observational Data for Integrated Maternity Care: Experiences with a Data-Infrastructure for Parents and Children in the Netherlands
Source: Int J Integr Care. 2023 Dec 20;23(4):20. doi: 10.5334/ijic.7012 (PMC10742107; doi:10.5334/ijic.7012)
Supplement: Supporting information. — Appendix with detailed information on 1) alignment with FAIR principles; 2) checks for selectivity within DIAPER and 3) the codebook of DIAPER. [file ijic-23-4-7012-s1.pdf]

Supporting information

S1: Alignment with the principles for FAIR data

The 'FAIR Guiding Principles for scientific data management and stewardship' were taken into account for the design and data management plan of DIAPER [10]. The FAIR principles provide a guideline to enhance the Findability, Accessibility, Interoperability and Reuse of data. The system and platform provided by SN supports the delivery of FAIR data to other researchers, who meet the strict requirements to access the safeguarded environment of SN in which DIAPER is located.

Findable

DIAPER and details on its contents are findable through the fully catalogued, open, online codebook and searchable through open standards and integration with existing services within SN. SN provides detailed information on its services and available data on their website [23]. The researchers that have designed DIAPER are also committed to spreading the word on the existence and potentials of DIAPER (e.g. through papers and presentations) and devote a notable portion of their time to providing other researchers with information on DIAPER.

Accessible

The data-infrastructure is explicitly built to offer access to all interested researchers. The researchers that have developed it are also committed to assisting interested researchers in the application process to gain access to DIAPER within the RA environment of SN and to provide them with the necessary tools and information to get started with the data after access has been granted. The SN platform makes a significant contribution to the accessibility of DIAPER, because it offers a convenient environment for multiple researchers to work in, at the same time, from different locations and on different studies. Technically speaking, gaining access to DIAPER is simple. The only potential barrier is in the legal process, because researchers do need to substantiate on what grounds they should be granted access, in the interest of data and privacy protection. The procedure to gain access to the RA environment of SN is outlined on their website [23].

Interoperable

In principle, DIAPER can be linked to any cohort or survey study, as long as this is in compliance with the GDPR (General Data Protection Regulation) guidelines [24]. SN takes care of pseudonymizing all (external) datasets by replacing any identifying variables by the 'Record Identification Number' (RIN), which is also included in the SSD. Through this identifier, different datasets can be linked. Therefore, SN has to able to properly assign a RIN based on other information in the data. Currently, within

DIAPER data from several different sources is already linked: Vektis, Perined and SN (several different sources of the SSD files). Several options are explored to add data from new sources, such as additional cohort studies. Next to this, the researchers that have developed DIAPER are in continuous contact with SN to improve the linkage procedures and possibilities. Additionally, the data within DIAPER can be accessed through a variety of different statistical software programs and packages.

Reusable

The entire foundation of DIAPER is set on the idea that it serves multiple purposes. DIAPER was never designed to answer one research question or to be used in a single project. Its contents and the platform on which it is located also contribute to its continuous reuse. On top of this, ODISSEI (Open Data Infrastructure for Social Science and Economic Innovations), the national research infrastructure for the social sciences in the Netherlands, embedded in the secure data environment of SN, acts as guarantor that the data facility remains accessible and reusable for future research including questions reaching far beyond the scope of the current applications. Extensive metadata to accommodate reuse is both documented in the DIAPER codebook and available on the SN website [23].

S2: Checks for selectivity of the sample in DIAPER compared to the general population

	% (number) births				
Year	2015	2016	2017	2018	Total
Statistics Netherlands	100% (168.184)	100% (170.323)	100% (167.595)	100% (166.331)	100% (672.433)
DIAPER*	93% (156.355)	93% (158.933)	92% (154.245)	88% (147.082)	92% (616.615)

**Level**	**Variable**		**Total births DIAPER** (n = 616.615)		**Total births SN** (n = 672.433)	
			**N**	**% / mean (sd)**	**N**	**% / mean (sd)**
**Mother**						
	Age			31,2 (4,734)		31,2 (4,791)
	Level of education					
		Low	26.204	4,3	29.684	4,2
		Middle	271.531	44,0	298.008	41,8
		High	246.618	40,0	272.536	38,3
	Ethnicity					
		Dutch (without migration background)	427.340	69,3	469.955	66,0
		Other western	65.773	10,7	80.627	11,3
		Non-western	123.502	20,0	148.096	20,8
**Father**						
	Age			32,5 (9,580)		34,2 (5,810)
	Level of education					
		Low	26.017	4,2	30.084	4,2
		Middle	265.213	43,0	293.215	41,2
		High	196.070	31,8	218.412	30,7
	Ethnicity					
		Dutch (without migration background)	417.169	67,7	461.220	64,7
		Other western	54.032	8,8	66.301	9,3
		Non-western	116.863	19,0	139.480	19,6
**Family**						
	Standardized household income (in percentiles)					
		1-20	98.327	16,0	110.656	15,5
		20-40	74.881	12,1	82.712	11,6
		40-60	109.845	17,8	120.939	17,0
		60-80	151.487	24,6	165.895	23,3
		80-100	171.685	27,8	188.672	26,5
	Household composition					
		Single parent household	74.852	12,1	82.262	11,6
		Family with both parents	524.777	85,1	578.744	81,2
		Other multiperson	4.814	0,8	5.347	0,8

Level	Variable		Total births DIAPER (n = 616.615)		Total births SN (n = 672.433)	
			N	% / mean (sd)	N	% / mean (sd)
		household				
		Institutions and (care) homes	1.759	0,3	2.493	0,4

Table: Results of the linkage process of DIAPER

**Linked in DIAPER means that a record has been identified in both Perined, Vektis as well as SN.*

S3: Codebook

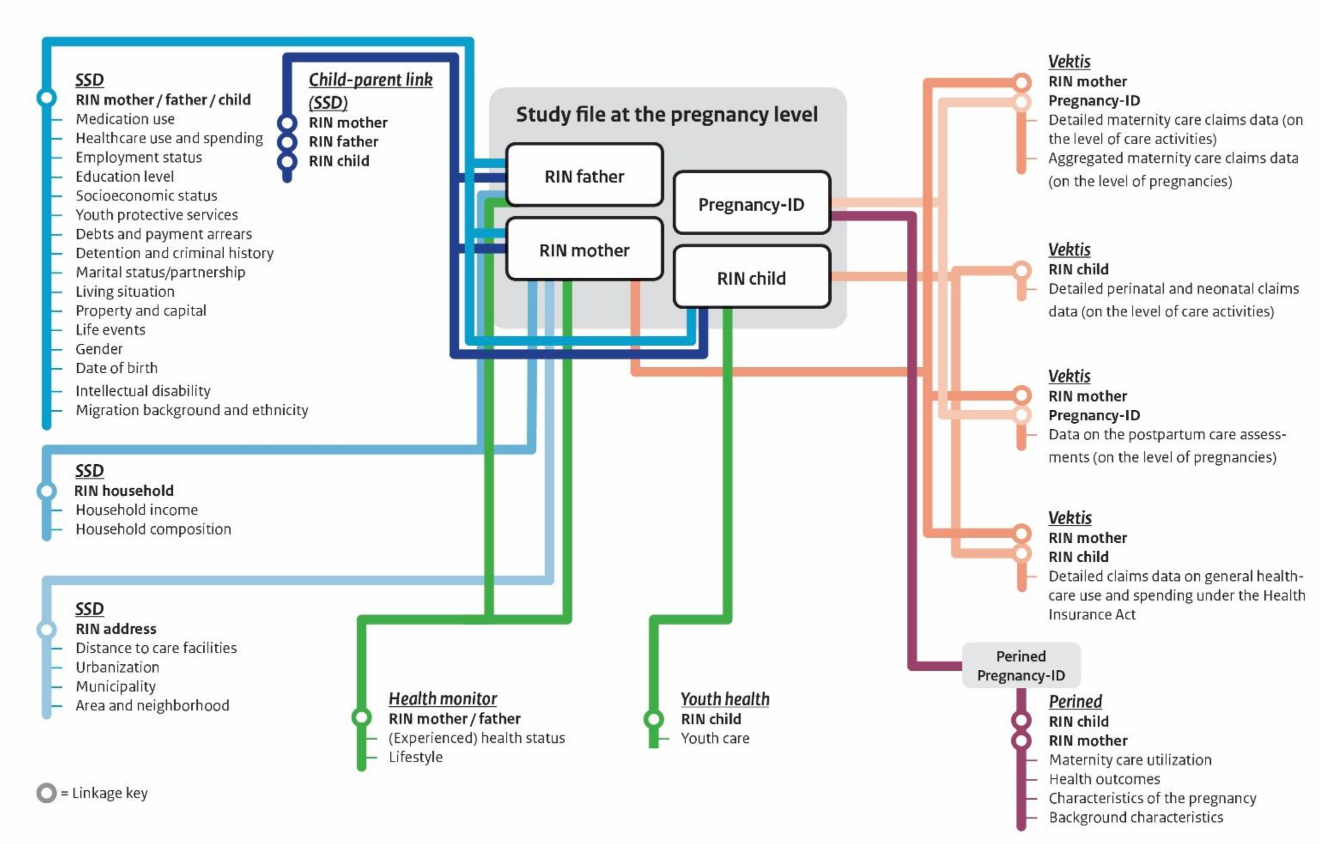

The figure above (included in the main text as part of Figure 2) gives a general overview of DIAPER's data sources and the available files and variables including employed linkage key. Please see Codebook DIAPER | RIVM for the complete and detailed codebook of DIAPER (in Dutch). This codebook is designed for and aimed at researchers who are working with DIAPER or interested to do so in the future and want to explore the possibilities regarding the available data. Existing code for DIAPER can be found on Github: https://github.com/rivm-syso/diaper.

For questions regarding (technical) details of DIAPER and/or the codebook please contact the authors through diaper@rivm.nl
